# ‘When I came to university, that’s when the real shift came’: alcohol and belonging in English higher education

**DOI:** 10.1080/13676261.2023.2190013

**Published:** 2023-03-20

**Authors:** Laura Fenton, Hannah Fairbrother, Victoria Whitaker, Madeleine Henney, Abigail Stevely, John Holmes

**Affiliations:** a School of Health and Related Research, University of Sheffield, Sheffield, UK; b Health Sciences School, University of Sheffield, Sheffield, UK

**Keywords:** Young people, alcohol, universities, belonging, friendship

## Abstract

While young people’s alcohol consumption has fallen sharply in the United Kingdom and other high-income countries, universities remain places where heavy drinking is routine and normative. Drawing on interviews with undergraduate students, this article explores how heavy drinking is part of how students negotiate a sense of belonging and form personal relationships. Theoretical work on belonging and relationality is used to make sense of students’ encounters with alcohol. Consistent with the decline in youth drinking, several interviewees had limited experience of heavy drinking prior to university, and some were not interested in taking it up. After describing how heavy drinking facilitates belonging in certain spaces of student life, we examine the strategies of non- and low-drinking students in navigating these spaces. Attending to their strategies suggests that becoming known as ‘social persons’ is key to negotiating belonging without drinking heavily. We conclude by considering how universities might better accommodate the desire for belonging for the increasingly large proportion of students with limited experience of or desire for alcohol by creating opportunities for students to form personal relationships in ways that do not involve alcohol or where alcohol is peripheral to the activity.

## Introduction

Young people’s alcohol consumption has declined across most high-income countries over the past two decades (Kraus 2018). Between 2003 and 2018, the proportion of 11–15 year-olds in England who have ever drunk alcohol dropped from 61% to 44%, while the proportion of those who reported drinking in the last week fell from 25% to 10% (NHS Digital 2019). Survey data further suggest that in England the decline in youth drinking is persisting as members of current youth cohorts enter adulthood, with the proportion of 16–24 year-olds who report drinking alcohol in the last week falling from 67% in 2002 to 41% in 2019 (NHS Digital 2020). A variety of possible causes have been proposed to explain the decline, including changes in parenting and the rise of internet-based technologies (Pape, Rossow, and Brunborg 2018; Törrönen et al. 2019). Qualitative studies have focussed on the significance of changes in youth culture, such as shifting socio-cultural norms and values around health, selfhood and authenticity (Caluzzi, MacLean, and Pennay 2020; Caluzzi et al. 2021). Overall, it is argued that alcohol no longer holds the same status as it did previously in youth culture, but rather has ‘lost its unquestioned symbolic power as a cool activity and rite of passage signalling entry to adulthood’ (Törrönen et al. 2019, 19).

Despite this decline, a persistent culture of heavy drinking among university students remains a public health and safety concern for universities and wider society (Conroy, Morton, and Griffin 2021), particularly given its potentially negative consequences for students’ health and wellbeing and increased risk of injury (Davoren et al. 2016). While those entering universities over recent years come from cohorts of young people who drink less than previous generations, studies demonstrate that drinking at harmful levels remains both normative and widespread among many students (Conroy, Morton, and Griffin 2021; Craigs et al. 2011; Davoren et al. 2016). The tension between the decline in youth drinking and persistently heavy student drinking requires examination (Conroy, Morton, and Griffin 2021, 2).

In this article, we investigate the continued role of heavy drinking in how students negotiate a sense of belonging against the backdrop of the wider decline in youth drinking. We explore how heavy drinking at the start of university is geared towards forging a sense of belonging. It occurs within specific spaces of student life – including student housing and student societies – that are central to what it means to be a student. Those students who do not drink heavily have to navigate the start of university and these spaces of student life carefully in order to negotiate a sense of belonging without drinking. Providing opportunities to negotiate belonging for the increasingly large proportion of students with limited experience of or desire for alcohol has become an important task for universities in countries like the United Kingdom (UK) that have experienced large decreases in youth drinking.

## Literature review

Despite the ongoing decline in youth drinking, university campuses in the UK have retained their reputation as ‘intoxigenic environments’ (McCreanor et al. 2008) in which ‘excessive alcohol consumption is viewed as relatively typical and acceptable’ (Conroy, Morton, and Griffin 2021, 2). While repeated, nationally representative surveys of university students’ drinking do not exist in the UK, a recent survey conducted by the National Union of Students (NUS) provides support for the claim that heavy drinking remains commonplace among a significant minority of students. A quarter of respondents reported drinking alcohol with the intention of getting drunk at least once a week (25%) (NUS 2018).

A wide range of explanations exist for why young people, including students, engage in heavy drinking. Several studies have explored the role of risk-taking in the transition to adulthood (e.g. Measham 2002, 2006), and how participating in a ‘culture of intoxication’ (Measham and Brain 2005) is tied up with constructions of identity (e.g. Hepworth et al. 2016), friendship practices (e.g. Niland et al. 2013; MacLean 2016), and the pursuit of ‘hedonistic’ pleasure (e.g. Measham 2004, 2006; Szmigin et al. 2008). Many of these studies treat students as a subset of the wider category of young people and do not address in depth how the unique relational, temporal and spatial features of student life shape their experiences with alcohol. Other research that focusses on student drinking suggests that attending to these features sheds light on its nature and prevalence. In terms of its relational dimensions, drinking is a practice that is oriented towards forging connections within groups. Drinking together is understood to be a significant part of how students form social bonds with one another (e.g. Hepworth et al. 2018; Brown and Murphy 2020). It is understood as simultaneously ‘liberating, pleasurable and a fun activity’ that is ‘central to belonging at university’ and a ‘socially expected part of university life’ that involves ‘pressure to consume and risk of social exclusion’ for not consuming (Supski, Lindsay, and Tanner 2017, 232). Temporally, the transition to university is a particularly significant time for social bonding, and thus for heavy drinking (Seaman and Ikegwuonu 2010; Hepworth et al. 2018; Brown and Murphy 2020). First-year students are often ‘recruited’ into the practice during orientation week (Supski, Lindsay, and Tanner 2017), but in many cases, alcohol consumption subsequently declines in the second and third years of study (Bewick et al. 2008). In relation to its spatial dimensions, student housing is a key site for pre-drinking^1^ and other alcohol-related socialising (Leontini et al. 2015, 2017; Supski, Lindsay, and Tanner 2017; Hepworth et al. 2018; Brown and Murphy 2020).

As alcohol facilitates social connection among students, non- and low-drinking students may become excluded from or marginalised within peer and friendships groups because they do not participate in drinking at the start of their first year when connections are formed (Brown and Murphy 2020). In Brown and Murphy’s (2020) study, the small number of students who did not drink or who drank lightly in their sample found forging connections to be more challenging. These students experienced ‘barriers to social connectedness as a result of [not drinking], including the feeling of having to explain non-participation’ (223). They found that it took longer to form peer groups and were more likely to experience initial difficulties in establishing friendships. They planned tactics, like finding a part-time job, for Freshers’^2^ week, knowing that they were likely to be excluded from typical activities. The authors argue that these types of ‘adaptations illustrate the potential for the alcohol-intense Freshers period to socially exclude some new students, with implications for their experience of transition’ (Brown and Murphy 2020, 221).

Looking beyond the initial transition to university, a growing literature explores the identity constructions of non- and low-drinking students, and how such students manage social situations involving alcohol (e.g. Herman-Kinney and Kinney 2013; Conroy and de Visser 2013, 2014; Banister, Piacentini, and Grimes 2019; Conroy, Morton, and Griffin 2021). Building on earlier work, Banister, Piacentini, and Grimes (2019) investigate how non-drinking students resist the identity of the ‘non-drinker’. Along similar lines, Conroy’s sole and joint-authored work takes a particular interest in how abstinent students explain their decision not to drink to others, particularly when they cannot ‘fall back on’ culturally accepted reasons, such as medical or religious reasons. These studies offer significant insight into the identity work and experiences of non- and low-drinking students. However, by treating such students as an exception – a ‘special’, if increasingly large group of young people – this literature risks missing the potentially transformative nature of the decline on *all* students’ drinking. It is not simply the case that there are likely to be more recent and incoming students who avoid alcohol. It is also true that incoming students, including those who do drink, are likely to have less experience overall with alcohol than preceding cohorts and are part of a generation where alcohol has a diminishing importance (Månsson, Samuelsson, and Törrönen 2020; Caluzzi et al. 2022).

Given that heavy drinking among students is oriented towards generating a sense of belonging and social connection, we next draw on theories of belonging and relationality as a conceptual backbone for interpreting the empirical findings presented in this paper.

## Belonging and relationality

The concept of belonging has two general meanings: a social meaning, in which it refers to an attachment to a particular group (e.g. families, communities), and a spatial meaning, in which it refers to an attachment to a place (e.g. homes, cities) (Gilmartin "belonging"). In the context of higher education, belonging has been defined as an ‘emotional attachment’ to one’s university, which involves ‘feeling at “home”, feeling safe’ (Gravett and Ajjawi 2021, 2), and has been principally explored in relation to retaining students within higher education (Thomas 2012, 2015). Moving away from ideas of belonging as ‘a fixed state of being’, Gravett and Ajjawi’s (2021) recent reconceptualization of belonging position it as a ‘relational and situated process’. This is because ‘we are always renegotiating our sense of belonging depending upon our context and what interaction we are having’ (Gravett and Ajjawi 2021, 6). Approaching belonging in this way draws attention to its fluidity. In Gravett and Ajjawi’s (2021) account, elements of the material environment play an important part in shaping belonging. For our purposes, this enables an appreciation of the active role of alcohol – as a mood-altering and inhibition-lowering intoxicant – in creating conditions for the negotiation of belonging (Fraser and Moore 2011; Bøhling 2015). In relation to spatial dynamics, in much research on belonging space is not understood as a ‘neutral container’ (Gravett and Ajjawi 2021, 5) or ‘passive backdrop’ (Jayne, Valentine, and Holloway 2008, 221), but rather comprises a ‘rich and fluid constellation of interactions’ (Gravett and Ajjawi 2021, 2; Massey 2005). Space is ‘always in the process of being made’ (Massey 2005, 9). This is important because it raises the possibility of spaces becoming collectively redefined by the actors inhabiting them. Intoxigenic environments, like other spaces, are open to change and redefinition. Indeed, through the prism of Massey’s understanding of space, we can see how the decline in youth drinking may mean that new cohorts of lighter drinking students collectively redefine the intoxigenic spaces of university life.

In exploring belonging, we draw attention to how personal relationships can attenuate the impact of social norms around drinking. The relational turn, as developed in the sociology of personal life (e.g. Smart 2007; May 2011), highlights how social life is navigated in and through personal relationships. In May’s terms, one’s ‘sense of self’ is ‘*constructed in relationships with* others, and *in relation to* others and to social norms’ (2011, 7; emphases original). The influence of social norms, like norms encouraging heavy drinking, is therefore attenuated by the more immediate context of our connections with others. The work of Finch (1989) and Finch and Mason (1993) approaches relationships as ‘developing in open-ended processes, not according to pre-existing rules’ (Roseneil and Ketokivi 2015, 144). It is a ‘history of interaction and reciprocity’ that generates ‘feelings of affection or obligation towards certain individuals’ (ibid). Ongoing relationships with others can be crucial in shaping how we respond to social norms. Drawing on this approach, we can see that relationships develop over time, as shared experiences and stories accumulate, and can take on a life of their own.

## Methods

In this article, we draw on interviews with 21 university students aged 18–25 conducted in 2019 as part of the Youth Drinking in Decline project, a mixed methods project exploring the decline in young people’s drinking. The project’s qualitative component adopted a multi-cohort research design to investigate continuities and changes in youth drinking and its intersections with broader youth culture. The project included 141 people across 83 interviews. Ninety-six young people aged 12–19 were interviewed in friendship groups of two to three people. The interviews were semi-structured and involved creative and participatory methods, including a relationship map and a visual elicitation technique. The relationship map was used to encourage young people to talk about how they spent their time and with whom, and how drinking and other health-related practices fit (or did not fit) into their everyday lives and relationships. Following arts-based workshops with young people, visual aids were created by a graphic designer that depicted different aspects of contemporary youth culture for the visual elicitation technique. The aids were shown to interviewees to elicit talk about their perceptions of the young people represented in the images, highlighting if they felt they would ‘fit’ into their friendship group. Interviewees were then asked to reflect on the health practices of the people represented. Forty-five young adults aged 20 to 35 were interviewed individually, using a semi-structured interview guide involving a timeline exercise. The timelines provided a basis for discussing topics, including how they spent their time as young people, the places they went and what was important to them. They were then asked for their views on the reasons for the decline in youth drinking. Interviews with both groups typically lasted in the region of 60 min.

This article draws on a subset of the qualitative dataset comprising 17 interviews with 21 participants. Seven first-year students were recruited as part of the cohort of young people. They consisted of five women and two men aged 18-19. All participants were White, except for one woman who was mixed White-Asian. Five were drinkers, while one was a light drinker, and another was a non-drinker. Interviews were conducted towards the end of the 2018/2019 academic year (March-May), meaning participants could reflect on their recent transition to university. Fourteen second and third-year students were interviewed individually as part of the cohort of young adults. This sub-sample consisted of nine women and five men. Ten participants were White; two were British Pakistani; one was Black African; and one was mixed British and South-East Asian. Ten were drinkers, and four were non-drinkers. All but one had drunk during their first year. Unlike the first-year students, who all lived in student housing, they had a variety of living arrangements, including living on their own, or with parents or partners.

Participants were recruited from a ‘redbrick’ and a ‘post-92’ university^3^ located in a city in Northern England, using internal university email distribution lists and advertisements on social media. We did not seek to recruit low-drinkers or abstainers, but rather to include broad representation of a range of drinking practices. Our sample reflects the typical breadth of drinking in a generation with substantially lower levels of alcohol consumption than its predecessors. Ethical permission was granted by the University of Sheffield. Pseudonyms are used throughout this paper.

Interviews were audio-recorded and transcribed for analysis. For the present analysis, transcripts of interviews with university students were analysed manually by the first author using thematic analysis (Ezzy 2002). Themes were then discussed with authors two and six. The centrality of heavy drinking to a sense of belonging in the key spaces of student life, and how this was negotiated by non- and low-drinking students, emerged as key themes. As mentioned, socio-cultural meanings and personal motivations surrounding heavy drinking beyond the negotiation of belonging have received extensive attention in literature on young people’s drinking, particularly the desire for ‘hedonistic’ pleasure (e.g. Measham 2006). While these meanings and motivations were discussed by several participants, they were less closely linked to their accounts of the place of drinking in their lives *as students*, that is, to their experiences in the spaces and temporalities specific to student life. Therefore, they are not focal points of the present analysis, though it is noted when they have been raised by participants.

Given the wider project’s interest in the decline in youth drinking, we reviewed our thematic analysis to explore what the data revealed about experiences with alcohol before, during and after the first year of university to better understand the issue of change over time in young people’s drinking. In what follows, we use this chronology to structure the presentation of our analysis, beginning with participants’ accounts of entering university as an entrance into episodic heavy drinking, moving onto experiences of heavy drinking in first year in different spaces of student life, and lastly considering accounts of transitioning out of heavy drinking after first year.

## The transition to heavy drinking in the first year of university

Starting university was not just an important educational transition but also a significant point of transition in the drinking careers of most interviewees. Prior to university, most had tried alcohol with parents and/or groups of friends, usually at house parties. Most had turned 18 prior to starting university and many of these had drunk in pubs and nightclubs. However, consistent with recent cohorts of young people in the wider population, several had limited experience with heavy drinking. The drinking occasions they encountered upon starting university were of a different nature. Reflecting on the experience of encountering ritualised and normalised heavy drinking as a first-year student, Brendan, a White British student at the redbrick university, commented:

I think if you're a non-drinker and got to university, you'd suddenly go ‘oh flip, like, this is a whole other world’ […] people do drink to excess at uni and that perhaps is more ‘this is how we roll’. And so, yeah, freshers, you are surrounded by people who are wanting to get smashed.

Before entering university, Brendan had drunk moderately at home with his parents. His parents had also given him cans of beer to take to get togethers at friends’ houses. Once starting university, he began ‘pre-drinking’ spirits with flatmates followed by going to nightclubs. This practice continued into second year when he moved into a shared rental house. Thus, the main shift in Brendan’s drinking habits that was occasioned by the move to university was the introduction of routine heavy drinking for the purposes of intoxication.

Hamza, a British Pakistani student, also at the redbrick university, remarked that the start of university was when a ‘real shift’ in his drinking began. Educated in Pakistan until he was 17, but with regular visits to the UK to see family, Hamza had started drinking towards the end of his time at school in Pakistan. Despite practising Islam, he and fellow students would ‘pay someone’ to purchase alcohol for them. However, it was after starting university in the UK that his drinking escalated:

I made a lot of international friends […] I remember one of the most drunk times in my life has been because of a Korean guy, like, he showed me this drink that they drink in Korea and I had, like, I hammered back about ten or twelve of those and I was utterly smashed, like, I fell over into the bushes, that’s how bad it got […] that’s when it really escalated when I started to come to uni.

Entering university marked the start of nights out. This was a significant shift in his drinking practices, as it signalled the start of the kind of ‘determined drunkenness’ discussed widely in the alcohol studies literature (Measham 2006).

Like Brendan, Hamza and several others, Alice first encountered ritualised heavy drinking as a ‘fresher’. A White British student at the redbrick university, she had experience of drinking with friends and co-workers prior to university, but the drinking at university was of a different order:

I’d say the first year was a bit of a shock to the system, purely because I’d gone from I guess such a sheltered sort of childhood, where people didn’t really drink a lot, they kind of drank for a bit of a social gathering. […] And then I get to uni first year, and it was like ‘Whoa!’ It was like people were drinking so much more than that I’d seen people drink … 

For Alice, like several others, starting university meant becoming involved in scenes of heavy drinking. Though shocked by what she saw, Alice enjoyed participating in pre-drinks and night outs with flatmates, as well as the occasional socials organised by her degree course’s student society. This was also the case for Brendan and Hamza, who also spoke about the pleasures of nights out with fellow students. Alice’s mention of flatmates and student society socials in her interview is telling, as these correspond to the two university-based spaces of student life that are frequently discussed in accounts of heavy drinking. We now turn to a consideration of these different spaces and how students negotiate belonging within them.

## Negotiating belonging with and without heavy drinking in first year

In what follows, we explore interviewees’ accounts of drinking in the contexts of pre-drinking rituals held in student housing and at social events organised by student societies, including course societies and sports teams. We then attend to the strategies of light and non-drinking students in navigating these spaces. While most students also spoke of drinking in pubs, bars and nightclubs in the city’s night-time economy, we focus here on drinking in spaces that are closely connected to the university as an institution. These spaces are by no means identical, either in general terms or in relation to their drinking norms. While drinking heavily on certain occasions was understood by most participants to be expected of students living in student housing, as well as those involved in some non-sport student societies, the pressure to drink heavily at the social events organised by sports societies was perceived to be far greater. This is not surprising given the abundance of research documenting cultures of heavy drinking in university sports societies (e.g. Partington 2013).

### Student housing

Most interviewees lived in purpose-built student housing in their first year. This usually consisted of apartments with a small number of private bedrooms, a shared kitchen and another communal area. Pre-drinking prior to going to nightclubs was the main form of drinking together within student housing. Consistent with other studies, drinking with flatmates was seen as enabling bonding in ‘a situation where rapid adaptation and acquisition of peer networks is prioritised’ (Brown and Murphy 2020, 225; Tinto 1975; Buote et al. 2007; Supski, Lindsay, and Tanner 2017).

Melanie, a White British student at the post-92 university, lived in a student flat during her first year. Her living arrangements were central to the start of her heavy drinking at university:

I first started drinking with my flatmates, because obviously it’s Freshers’ week. […] I mean it was what everyone did – my entire flat went out. […] we’d like get stuff from [a shop] like the cheapest kind of stuff you can find, get quite drunk at pre drinks, sometimes play games […]. And then we’d go out to a club … .

Melanie’s account suggests an inevitable link between heavy drinking and the start of university (‘because obviously it’s Freshers’ week’). When asked if her motivation on a night out was to ‘get drunk’, Melanie replies:

Mainly – partly because everyone else was doing it. […] I think it was obviously because you’re just lumped in with people that you don’t know. And it’s hard to be a bit different. And also like, pretty much the only time they would ever meet up was to drink. So if I wanted to make friends with them I kind of had to drink … 

Melanie appears to have an internalised the idea that drinking with flatmates was an obligation, an expectation that she must live up to in order to ‘fit in’ and negotiate acceptance within the group. Consistent with Brown and Murphy’s study (2020), the transition to university for many first-year students living in student housing involved them feeling as though heavy drinking was not so much a choice but a necessity if one was to make friends. This was tied to being ‘lumped in with people that you don’t know’, living together in close proximity. The intensity of relationships with flatmates was commented on by others. In the words of Henry, a first-year student of White sub-Saharan African origin at the redbrick university, ‘you’re piled on top of each other’ so ‘you get to know each other quite well’. Interestingly, Henry had intended to give up drinking before starting university, but decided to wait after the Freshers’ period because he perceived drinking to be central to getting to know people. Negotiating social connections and belonging to the group was believed to necessitate participating in heavy drinking for Melanie, Henry and others, though in Henry’s case, the drinking ceased once social connections were established.

Edith, a first-year student at the redbrick university, was an exception in that she lived in student housing, participated in pre-drinks and negotiated a sense of belonging, but did so without drinking heavily. Edith speaks in negative terms about alcohol in her interview, stating that she hates the taste of alcohol and the risks to personal safety that she believes arise when one becomes intoxicated. She comments: ‘I seriously don't see the appeal of [drinking heavily], I think it’s damn stupid!’ She was interviewed alongside her flatmate, Greg, and another first-year student, Bea, who both speak in far more positive terms about alcohol. Edith was close to her flatmates, who she described as a ‘really key part of my social life’. She associated her shared flat with a sense of security and belonging, as ‘somewhere that I can go home and relax and they’re there and I can talk to them and it’s nice, but then if I want some time I can just go to my room’. Edith’s dislike of and low tolerance for alcohol becomes an in-joke in the interview. When she told the interviewer that she does not drink much, Greg remarked ‘It’s funny when you do though!’ Bea joked that Edith’s pre-drinks are her ‘actual drinks!’ Edith tended to have one drink during pre-drinks, ‘usually a spirit and a mixer, just because that is all I can manage. I hate the taste of everything else … ’ Asked if it is important that she drinks during pre-drinks, the following dialogue ensued:

Edith: Erm..

Interviewer: Does everybody drink?

Greg: Pretty much.

Bea: Pretty much.

Edith: Pretty much, yeah.

In the interview, having one drink emerges as a sort of bargain Edith makes to socialise with flatmates during pre-drinks. Edith’s dislike and low tolerance for alcohol is responded to playfully by the other two interviewees and does not appear to act as a barrier to her feelings of ease and social connection with her flatmates. We see here how in contexts where students feel a sense of belonging and are known to others as individuals as opposed to simply ‘non-drinkers’, perceived pressures to drink heavily may be weaker or may be suspended altogether.

### Student societies’ socials

Like student housing, student societies were described as an important source of community and belonging. It was, therefore, problematic for some that societies’ social events involved heavy drinking and that this drinking could feel as if it was obligatory if one was to make friends. While the socials of some course-based societies were often described as nights out involving heavy drinking, this was not always the case, with some participants describing their course-based socials as calmer, less alcohol-centric social events. They sometimes included days out and trips, such as walks around nearby countryside and visits to the theatre. Moreover, the pressure to drink heavily at non-sport societies’ social events was not represented as being anywhere near as strong as the pressure to do so at the events of sports teams. Sports teams’ weekly socials were synonymous with heavy drinking. Negative media coverage of the excesses of initiation rituals organised by student sport teams was mentioned by a few participants. Following media coverage of the death of a first-year student at the University of Newcastle from alcohol poisoning in late 2016, initiation rituals began to receive attention in the student press via the student website, *The Tab*.

Asked to describe his team’s weekly socials, Henry replied:

Honestly, [they’re] just a lot of drinking. I get it quite bad because I stopped drinking, so [they make me drink] nasty mixes of non-alcoholic things. But yeah, that will all stop next year when I’m not a fresher […] Apparently they’ve calmed it down a lot but there’s […] almost like a fresher bashing culture. […] you don’t like it but you stick around because you like [the sport].

‘Fresher bashing rituals’ had created a context in which it was especially difficult for first years to refuse alcohol. This is echoed by Peter, who reflects on sports teams’ socials he attended as a first-year student at the post-92 university:

… that was probably the biggest shock […] after games – […] this thing called Amy Winehands, where you sellotape a bottle of wine to your hand and – yeah. So it’s that excessive […] but because it was 40 odd people in exactly the same state, it seemed acceptable, because you’re together in it, so it’s fine. […] sports teams are a big culprit of the bad culture at uni. A lot of bad stuff happens, but I think that is changing.

For Peter, the collectively legitimated nature of heavy drinking during initiation rituals makes the consumption of harmful and even dangerous amounts appear ‘acceptable’. Both Henry and Peter mentioned that the situation had improved over recent years, with the latter having first-hand experience of witnessing changes.

Sports clubs were typically gender-segregated and experiences of negotiating belonging were similarly gendered. For example, women interviewees involved in sports did not mention initiation rituals. However, like the young men they very much associated clubs’ socials with heavy drinking. Melissa, a White British student, and her flatmate, Alexa, a mixed White-Asian student, were both first-year students at the redbrick university. As members of the same student sports team, they spoke positively about weekly nights out with the club, which involved a ‘bar crawl and then we end up in [in a night club]’. However, Caroline, a third-year student of Black African origin at the redbrick university, lamented the fact that her team’s socials involve heavy drinking. While Caroline does drink, she does not like ‘binge drinking’:

… every week there’s a very heavy drinking social. And I know that’s the case in a lot of sports clubs. […] I don’t enjoy binge drinking, I don’t enjoy activities where you basically have to be drunk to enjoy them. I found it like hard – like I didn’t fit in very well within the [sports] club because I didn’t go out and drink with them a lot.

Caroline’s sense of ‘fitting in’ with fellow members of her club has thus been stymied by the club’s reliance on heavy drinking as the main means of socialising. Furthermore, she saw the link between heavy drinking and sport as intimately tied up with who ‘belongs’ at university:

If I was to describe a student who drank a lot, I would probably think a white male, who probably plays rugby. I know it’s a bit of a stereotype […] when you think of universities the people most represented are White home students, who are probably middle class and privately educated.

She later stated that she ‘often felt excluded’ from her team both for not drinking heavily and because of her ‘African heritage’, commenting that this sense of exclusion extends to ‘the Students’ Union as a whole’.

For others who do not drink at all, navigating sports clubs’ socials posed challenges. Colin, a White British student who had drunk on only one occasion prior to university, stopped participating in student clubs because of the heavy drinking:

I was a bit disappointed […] I joined [a sport] Society and, like, there’s such a big emphasis on nights out […] there’s a [sports team] tour thing, like, I think everyone has a tour going on, like, every Department has one, yeah, ‘come out, this is cheap here, like, the alcohol’s cheap, blah blah blah, go to a club every night’ […] I didn’t expect it, but there’s so much of an emphasis on drinking … 

The fact that social events for sports teams, and in Colin’s case, for his course, were centred around heavy drinking meant that Colin chose not to participate in such events, with consequences for his sense of belonging at university.

Unlike Colin but similar to Edith, Henry continued to participate in social events involving heavy drinking despite giving up alcohol after Freshers’ week. As mentioned, he decided before starting university that he was going to stop drinking ‘just to be more healthy’ but subsequently decided to still drink during Freshers’ week because it’s ‘quite a big thing’. He described the challenges of not drinking at the socials organised for sports clubs in the months that followed:

We get quite a lot of people [at sports socials] that are like ‘oh, why don’t you drink? It’s a bit boring’ […] Even at my end of season meal, there’s a few people that offer me pints […] but you know, the majority […] are like ‘fair enough for sticking to your guns’. They just accept it and, you know, it’s not really a thing for like 95% of them. […] as long as the rest of the club’s there, [the 5% who give me a hard time] wouldn’t say anything. It’s more like when you’re on your own and you’re having to see them and they’re a bit funny but, you know, you just avoid those people really.

Henry’s account is revealing of the protective power of personal relationships when ‘peer pressure’ is exerted by a small minority. He navigates these spaces by avoiding this minority and ensuring he is surrounded by the more supportive, or at least indifferent, majority, particularly fellow members of his own club. To them, he is known as ‘Henry’, as opposed to ‘that guy who doesn’t drink’ (the label given to the flatmate of another interviewee). This navigation strategy is all the easier to enact when he is in the company of his flatmates:

It’s definitely easier to be a non-drinker when I’m out with my flat than it is when I’m out with [my sports team] […] [my flatmates aren’t] bothered either way. Like we do this thing you have to do 19 shots on your birthday. […] when I did mine they were just like, you know, Bovril or […] Yakult, whatever, literally whatever was lying around. So I just did mine, like you know, you just get on with it. But yeah, [with my sports team] […] it would be people saying ‘oh no, you have to have your shots though’. But yeah, they just don’t care whether you drink or not, it’s just do you come out, you know, are you social? So that’s the only thing they’re really bothered about.

According to Henry, his flatmates’ concern was that he was sociable, and not whether he drank alcohol, accepting shots of gravy or fermented milk as substitutes for spirits. The point was that he participated in rituals. That this is more strongly the case among his flatmates than his team suggests that the intensity of personal relationships – that is, both how well one is known as an individual and as a part of the group – are important in overriding the pressure to drink. It further suggests that the pressure to drink in student housing may indeed be limited for many to the period before they are known to their flatmates as individuals.

## Transitioning out of regular heavy drinking during and after first year

While some interviewees continued to drink heavily as they progressed through university, most found that the conditions encouraging heavy drinking in the first year were no longer in place. As discussed in the previous section, the conditions in the first year facilitating heavy drinking included living in shared housing with large groups of other students who they were in the process of getting to know, and joining student societies in order to meet other students. Those who continued the practice of routine heavy drinking after the first year tended to be people who had continued living with groups of other students in shared flats or houses, suggesting the potential significance of living arrangements.

Student trajectories through university can diverge after the first year, as some study abroad or take up work or voluntary placements. This means time away from the spaces of student life in which belonging is negotiated. More commonly, changes related to the structure of degrees at English universities help to explain why most participants reduced their heavy drinking as they progressed through university. The first year of a degree typically counts less, if at all, towards the final classification achieved for the degree. For many, first year involved experiencing periods with ample amounts of free time prior to and after coursework deadlines were met and exams were taken. Henry’s interview was conducted towards the end of first year. He remarked that ‘quite honestly I spend most of the time just socialising. […] I covered quite a lot of it in sixth form […] – especially the first half of the year – I’d covered a lot of it already’. By contrast, as a third year, Caroline stated that her ‘priorities are very much […] academic’.

Others similarly described a ramping up of the intensity of academic study after first year, with consequences for how they spent what leisure time they had and how they assessed the consequences of a ‘hang over’ for the next day. Like Henry, Melanie stopped drinking altogether in first year. Asked why, she explained: ‘I wasn’t particularly enjoying it. Like I would get really, really bad hangovers […] And obviously with all the work I had to do and the fact I was working as well […] [it] wasn’t something I had the time for anymore’. She feels less pressure to drink now that her first year is over, and believes that there are ‘a lot of people in second year who feel a lot less pressure to drink’. She explained why this is the case: ‘I think people get more invested in their studies, so they see it as less of an integral part of university life, when they actually have work to do’. She also moved in with her partner, who did not drink, which made avoiding alcohol easier. We can see how in Melanie’s case heavy drinking with flatmates was a temporary phase limited to the first months of her first year. Once friendships were formed and she had adjusted to her routines at university, heavy drinking began to feel optional and she was able to opt against it. Thus, as we have seen, less involvement in the spaces of student life in which heavy drinking oriented towards belonging is practised, and a reduced amount of leisure time stemming from the structure of degrees, mean that heavy drinking became either more occasional or was abandoned altogether by many in our sample as they progressed through their degrees.

## Discussion

Our findings suggest that even in the context of a large and long-term decline in youth drinking, heavy drinking at university is produced by students’ entrance into the different spaces of student life in which belonging is negotiated. This varies across the spaces, with sports teams’ initiation rituals emerging as spaces characterised by intense pressure to conform to heavy drinking norms. Many young people now enter university with little experience of heavy drinking, yet heavy drinking remains key to the formation of a sense of belonging and to the formation of personal relationships that may endure well beyond the years of university study. While several participants found heavy drinking to be pleasurable and welcomed its introduction into their lives, others were far less enthusiastic. These latter participants were concerned by how central heavy drinking was to forging friendships in first year. Abstaining or drinking lightly while socialising in intoxigenic environments like pre-drinks in shared accommodation and sports socials is ‘no small accomplishment’ (Conroy, Morton, and Griffin 2021, 2). However, drawing on the sociology of personal life, we can see how the cases of Edith and Henry illustrate that personal relationships in such contexts can override the pressure to drink. In other words, histories of ‘interaction and reciprocity’ can create affective ties that attenuate the impact of social norms (Roseneil and Ketokivi 2015, 144). Though Edith still felt compelled to have at least one drink, and Henry felt he had to wait until after Freshers’ week to stop drinking, by socialising with people to whom they are already known as, following Strathern (see Strathern 2020), social persons – named individuals, with biographies, capacities and relations that extend beyond themselves – these light or non-drinkers may not have to justify their decision to limit or avoid drinking.

The accounts of participants who do not drink heavily suggest that the key to reducing the pressure to drink in intoxigenic settings is to create opportunities for students to negotiate relationships and become known as social persons prior to when heavy drinking becomes a central mechanism of group formation and consolidation. Drinking is a ‘technology of friendship’ (MacLean 2016); its central role in the creation of friendship means that it is also a technology of belonging. Understanding how heavy drinking produces belonging affords possibilities to create opportunities for incoming cohorts of students with little or no experience of or interest in alcohol to negotiate belonging without having to practise heavy drinking. The decline in youth drinking and the rising numbers of students who abstain or moderate present universities with opportunities and responsibilities to rethink the provision of social activities beyond existing ways of operating (Brown and Murphy 2020, 226). At the university where Brown and Murphy conducted their study, the student union organised events advertised as ‘alcohol-free’ for Freshers’ Week for daytimes but not evenings, creating ‘temporal segregation’ between drinking and non-drinking students (223). Segregating drinkers and non-drinkers temporally or otherwise is limited as a way forward, particularly given that many students re-aggregate in one of the principal places where processes of bonding through heavy drinking occurs: student housing. A better way to address the issue is to provide opportunities for flatmates, course-mates and teammates to get to know one another during the early months of first year in ways that do not involve alcohol, but where there is no expectation that they would (e.g. day trips, walks in the countryside, cinema trips, etcetera). This practice was already in place among some course societies and was valued by some of our participants.

Returning to the challenge of interpreting the intoxigenic nature of student life against the backdrop of the decline, consistent with other studies (e.g. Supski, Lindsay, and Tanner 2017), we found that the first year of study appears to be an exception for many in terms of their involvement in routinised heavy drinking, though Bewick et al.’s (2008) study suggests that this was the case prior to the decline. It is possible that a large proportion are returning to their pre-university trajectory of alcohol consumption, an idea that is consistent with declining levels of drinking among adults in England (NHS Digital 2020)). While second- and third-year students’ retrospective accounts of drinking across time suggest that for many the first year may be well be a transitory stage in their drinking, the significance of these early months for the formation of a sense of belonging means that not participating in such rituals may bring with it consequences (Supski, Lindsay, and Tanner 2017; Hepworth et al. 2018; Brown and Murphy 2020; Conroy, Morton, and Griffin 2021). We see this with the case of Colin, who expresses disappointment at not being able to participate in much of his university’s social life. A lack of experience with alcohol amongst many in our sample means that many only ever ‘matured into’ heavy drinking in their first year. Crucially, the fast-paced nature of their introduction to heavy drinking does not have the characteristics of a process of maturation. Here, universities, including student unions, may need to build on existing efforts, like the NUS Alcohol Impact programme (NUS 2018), to address their role in introducing many young people with limited prior experience of alcohol to heavy drinking.

## Conclusions

In this article, we have argued that heavy drinking persists at universities in part because it is embedded in how students build social connections at a crucial juncture in their time as students, that is, in the initial months of their first year. Prior to starting university, many in our sample had limited experience with heavy drinking, a finding that is far from surprising given the decline in youth drinking that has been underway since the early 2000s. In their efforts to negotiate belonging, many first-year students enlisted alcohol to create social bonds and get to know one another in key spaces of student life, including student housing and the social events of student societies. Though several found their introduction to heavy drinking in these spaces to be pleasurable, this was not always the case. In navigating these spaces, some of the students who did not wish to drink heavily felt as though some level of drinking was required in order to participate in social bonding and to become known as social persons. This was especially the case in sports societies. After friendships had been formed and academic pressures intensified in second and third years, heavy drinking tended to become a less routine feature of their lives. To address and take greater responsibility for their role in introducing many young people to heavy drinking, universities might consider working with student housing providers and student unions to provide more opportunities for students to get know one another through events or occasions which are not labelled as ‘alcohol free’ but rather where alcohol is not central to the nature of the activity at hand.

## Notes

1 Pre-drinking refers to the practice of drinking in a group, usually in a domestic setting, in preparation for a night out.

2 ‘Freshers’ refers to those in their first year of university. Freshers’ week is the first week of the academic year when students do not normally have classes and student unions and societies organise events for new students. In Australia, the week is referred to as ‘orientation week’, or ‘O-week’ (Supski, Lindsay, and Tanner 2017).

3 In the UK, redbrick universities are universities that were founded in industrial cities in the nineteenth and early twentieth centuries. Post-92 universities are former polytechnics (institutions offering vocational training) that were reclassified as universities following the Further and Higher Education Act 1992.
